# Genome-Wide Association of Lipid-Lowering Response to Statins in Combined Study Populations

**DOI:** 10.1371/journal.pone.0009763

**Published:** 2010-03-22

**Authors:** Mathew J. Barber, Lara M. Mangravite, Craig L. Hyde, Daniel I. Chasman, Joshua D. Smith, Catherine A. McCarty, Xiaohui Li, Russell A. Wilke, Mark J. Rieder, Paul T. Williams, Paul M. Ridker, Aurobindo Chatterjee, Jerome I. Rotter, Deborah A. Nickerson, Matthew Stephens, Ronald M. Krauss

**Affiliations:** 1 Department of Human Genetics, University of Chicago, Chicago, Illinois, United States of America; 2 Children's Hospital Oakland Research Institute, Oakland, California, United States of America; 3 Statistical Application, Pfizer Global Research and Development, Groton, Connecticut, United States of America; 4 Center for Cardiovascular Disease Prevention, Brigham and Women's Hospital and Harvard Medical School, Boston, Massachusetts, United States of America; 5 Department of Genome Sciences, University of Washington, Seattle, Washington, United States of America; 6 Center for Human Genetics, Marshfield Clinic Research Foundation, Marshfield, Wisconsin, United States of America; 7 Medical Genetics Institute, Cedars-Sinai Medical Center, West Los Angeles, California, United States of America; 8 Department of Pharmacology and Toxicology and Department of Medicine, Medical College of Wisconsin, Milwaukee, Wisconsin, United States of America; 9 Life Sciences Division, Ernest Orlando Lawrence Berkeley National Laboratory, Berkeley, California, United States of America; 10 Department of Statistics, University of Chicago, Chicago, Illinois, United States of America; Dr. Margarete Fischer-Bosch Institute of Clinical Pharmacology, Germany

## Abstract

**Background:**

Statins effectively lower total and plasma LDL-cholesterol, but the magnitude of decrease varies among individuals. To identify single nucleotide polymorphisms (SNPs) contributing to this variation, we performed a combined analysis of genome-wide association (GWA) results from three trials of statin efficacy.

**Methods and Principal Findings:**

Bayesian and standard frequentist association analyses were performed on untreated and statin-mediated changes in LDL-cholesterol, total cholesterol, HDL-cholesterol, and triglyceride on a total of 3932 subjects using data from three studies: Cholesterol and Pharmacogenetics (40 mg/day simvastatin, 6 weeks), Pravastatin/Inflammation CRP Evaluation (40 mg/day pravastatin, 24 weeks), and Treating to New Targets (10 mg/day atorvastatin, 8 weeks). Genotype imputation was used to maximize genomic coverage and to combine information across studies. Phenotypes were normalized within each study to account for systematic differences among studies, and fixed-effects combined analysis of the combined sample were performed to detect consistent effects across studies. Two SNP associations were assessed as having posterior probability greater than 50%, indicating that they were more likely than not to be genuinely associated with statin-mediated lipid response. SNP rs8014194, located within the *CLMN* gene on chromosome 14, was strongly associated with statin-mediated change in total cholesterol with an 84% probability by Bayesian analysis, and a p-value exceeding conventional levels of genome-wide significance by frequentist analysis (*P* = 1.8×10^−8^). This SNP was less significantly associated with change in LDL-cholesterol (posterior probability = 0.16, *P* = 4.0×10^−6^). Bayesian analysis also assigned a 51% probability that rs4420638, located in *APOC1* and near *APOE*, was associated with change in LDL-cholesterol.

**Conclusions and Significance:**

Using combined GWA analysis from three clinical trials involving nearly 4,000 individuals treated with simvastatin, pravastatin, or atorvastatin, we have identified SNPs that may be associated with variation in the magnitude of statin-mediated reduction in total and LDL-cholesterol, including one in the *CLMN* gene for which statistical evidence for association exceeds conventional levels of genome-wide significance.

**Trial Registration:**

PRINCE and TNT are not registered. CAP is registered at Clinicaltrials.gov NCT00451828

## Introduction

Statins are the most widely prescribed drug class for the prevention of cardiovascular disease (CVD) and act primarily by lowering plasma LDL-cholesterol (LDLC) [1], [2], [3]. Statin-induced reductions of LDLC vary among individuals and this may reflect genetic differences [4], [5]. Variation in LDLC response has been associated with several loci involved in cholesterol and lipoprotein metabolism including *APOE, HMGCR, PCSK9, LDLR* and *APOB*, but these account for only a small portion of the variance in LDLC response [4], [6], [7]. Recently, a common nonsynonymous SNP in the gene encoding the cellular motor protein KIF6 was found to be associated with both increased CVD risk and greater statin-related CVD risk reduction but not with lipid or lipoprotein response to statins [8].

Genome-wide association (GWA) studies provide a more comprehensive approach for identifying genetic loci associated with statin response. GWA studies have identified many loci associated with plasma lipid and lipoprotein traits, including several not previously known to be related to lipoprotein metabolism [9], [10], [11] [12]. However, there is to date only one report of GWA of lipid response to statin treatment [13]. This involved ∼2,000 participants in the Treating to New Targets study who were treated with atorvastatin 10 mg/day for eight weeks. However, no SNPs were identified from this analysis that were convincingly associated with atorvastatin-mediated lipid changes (*P*>1×10^−7^ for all associations) [13].

The power of GWA studies to identify and convincingly document associations of SNPs with complex traits has been greatly enhanced by generating large data sets from combined studies [9], [10], [14], [15], [16]. Motivated by this, we performed a combined analysis of the three statin GWA studies currently available to us: TNT and two previously-unpublished trials. To deal with the fact that different studies typed different SNPs, we used imputation methods [17], [18] to infer genotypes for approximately 2.5 million HapMap SNPs in all three studies [17], [18], [19]. To allow for systematic differences among the three statin trials, we normalized phenotype measurements for each individual relative to other individuals within the same study before performing a fixed-effects combined-analysis. In addition we used a novel Bayesian statistical approach, with a bivariate phenotype derived from pre-statin and post-statin phenotype levels, to simultaneously identify both statin-independent and statin-dependent SNP associations.

## Methods

### Study populations

The 3,936 Caucasian individuals in these analyses had participated in one of three statin trials, all of which have been described previously [5], [20], [21].The Cholesterol and Pharmacogenetics (CAP) trial involved 944 healthy volunteers, 609 of whom were Caucasian, treated for six weeks with simvastatin 40 mg/day at two study sites (University of California, Los Angeles and San Francisco General Hospital) [5]. Blood samples were collected for lipid and lipoprotein analysis twice prior to treatment (screen and entry visits) and twice while on treatment (4 and 6 weeks). There were no significant differences between the two pretreatment measurements or the two post-treatment measurements, and therefore these values were averaged to obtain pre-treatment and post-treatment values, respectively.

The Pravastatin Inflammation/CRP Evaluation (PRINCE) study enrolled 1702 individuals with no history of prior heart disease and LDLC >135 mg/dL, and 1182 individuals with known cardiovascular disease (CVD)–defined as previous myocardial infarction, stroke, or coronary revascularization from 1143 clinical sites across the United States (≤4 participants/site). Participants were treated with 40 mg/day pravastatin for twelve weeks [20]. Individuals with no history of prior heart disease were treated as part of a randomized controlled double blind study and those with CVD history were provided with open label pravastatin. Our analyses were not stratified by treatment group assignment. Laboratory analyses were performed on blood samples collected once prior to treatment, and once following twelve weeks of treatment. 1056 participants also provided a blood sample following twenty-four weeks of treatment. For those individuals who provided two post-treatment samples, these values were averaged to obtain a single post-treatment value. DNA samples were collected from 1536 PRINCE participants including 1362 that were self-identified as Caucasian [22].

The Treating to New Targets (TNT) study followed 10,001 patients with clinically evident CHD and with LDLC 130 to 250 mg/dL at screen and ≤130 mg/dL following 4 weeks on treatment [21]. CHD was defined by previous myocardial infarction, previous or current angina with objective evidence of atherosclerosis, or history of coronary revascularization. Lipids were analyzed using blood samples collected once prior to treatment and once following eight weeks of treatment. Individuals with evidence of poor compliance, assessed on the basis of LDLC >130 mg/dL following treatment for 4 weeks, were excluded from analysis. 2,092 patients of European ancestry were selected for inclusion in whole genome genotyping and these included 523 individuals who had coronary events during the trial (“cases”) and 1,569 selected “control” individuals who did not. Controls were matched 3∶1 to cases by ancestry, age, gender, smoking, diabetes, hypertension, baseline glucose, and screening LDLC [13]. Of the selected individuals, genotyping data was successfully obtained on 1,976 (call rate ≥80%).

Approvals for each study were obtained from the Institutional Review Boards at participating institutions for that study and each participant signed a statement of informed consent that provided permission for samples to be used in future genomic studies. All three studies measured LDLC, total cholesterol (TC), HDL-cholesterol (HDLC) and triglycerides (Tg) in laboratories certified by the Centers for Disease Control and Prevention (Atlanta, Georgia).

### Genotyping and Genotype Imputation

Whole-genome genotypes for CAP and PRINCE participants were measured in two stages (henceforth referred to as Stage I and Stage II). In Stage I, 304 CAP and 675 PRINCE participants were genotyped for 314,621 single nucleotide polymorphisms (SNPs) selected to tag common genomic variation in Caucasians (HumanHap300 bead chip, Illumina, San Diego, CA). These SNPs were derived from Phase I+II of the International HapMap Project (www.hapmap.org) to tag common genomic variation across individuals of European decent. This platform provides genomic coverage in Caucasians of all Phase I+II loci of 91% for an r^2^ threshold ≥0.5 and 80% for an r^2^ threshold ≥0.8 [23]. Our analysis of genotyping quality, using ∼18,000 SNPs contained on the HumanHap300 bead chip, and a separate Human-1 (109,000 sites) bead chip showed a genotyping concordance rate of 99.97%.

In Stage II, 290 CAP and 687 PRINCE samples were genotyped. This included 280 CAP and 652 PRINCE samples (N = 932) that were genotyped at 620,901 sites using the HumanQuad610 bead chip (Illumina), which is also designed to tag common variation within individuals of European ancestry, and a partially-overlapping set of 292 CAP and 634 PRINCE samples that were genotyped at 12,959 sites using a custom-made iSelect chip (N = 926). The average call rate for stage II genotyping was 99.8%. Four samples were excluded from further analysis based on gender discrepancies. Concordance between SNPs on the HumanQuad610 bead chip and the custom-made iSelect chip exceeded 99.5%.

Genotyping in TNT participants was performed as described previously [9], [13]. Genotypes were assessed for 322,185 SNPs using the Perlegen platform (Perlegen, Mountain View, CA). Homozygous sites and SNPs with call rate <80% were eliminated from analysis, leaving 291,988 SNPs. Of the 2092 participants selected for analysis based on clinical characteristics, genotyping was performed on the 1984 with sufficient quantity and quality DNA. Of these, genotyping on eight individuals was omitted from analysis due to sample mishandling. For the 1976 remaining samples, sample call rates were all >91% and the average SNP call rate was 97.9%.

We used the genotype imputation software BIMBAM [18], [19] to infer genotypes for each individual at the approximately 2.5 million SNPs typed in the HapMap (phase II) CEU parents [23]. Imputation procedures rely on the patterns of correlation among typed and untyped SNPs inferred from HapMap individuals to estimate genotypes at the untyped SNPs in each individual. Imputation has the benefits of both maximizing genomic coverage and facilitating combined analyses of studies involving different genotyping platforms. All analyses were limited to Caucasians.

### Statistical Analysis

We analyzed data from each of four phenotypes (LDLC, TC, Tg and HDLC) separately. For each individual there are two measures of each normalized phenotype: one reflecting levels pre-statin exposure (baseline, X), and one reflecting levels post-exposure (on study, Y). The values of X and Y are derived from extensive normalization procedures designed to eliminate the potential for false positive associations due to systematic differences among studies (see below). However, it may aid interpretation of methods and results to note that in practice X is approximately the log of the pre-statin measure and Y is approximately the log of the post-statin measure (both centered to have mean 0).

In brief, our analyses aim to identify SNPs that are associated with the bivariate outcome (X,Y), and to distinguish between SNPs that affect both X and Y in the same way (which we term “statin-independent associations”) and SNPs that affect X and Y differently (“statin-response associations”). (Although this definition of statin-response associations may seem natural, there are some issues with this definition that deserve attention, as we return to in the discussion.) To do this it is convenient to reparameterize (X,Y) in terms of the derived “sum” (S) and “difference” (D) phenotypes, S = Y+X and D = Y−X. Because of the 1-1 correspondence between (X,Y) and (S,D), SNPs will be associated with (X,Y) if and only if they are associated with (S,D). But the (S,D) parameterization is convenient because i) statin-response associations are exactly those SNPs that are associated with D, and ii) S and D are uncorrelated.

For each SNP and phenotype, we assessed the fit of four different models: H_0_, the null model that the SNP is associated with neither S nor D; H_S_, the SNP is associated with S only; H_D_, the SNP is associated with D only; H_S+D_, the SNP is associated with both S and D. Note that H_D_ and H_S+D_ correspond to statin-response associations, whereas H_S_ corresponds to statin-independent associations. One might expect many previously-reported associations between genetic variants and lipid phenotypes to fall into this last category, providing a helpful check of the consistency of our results with previous lipid studies not involving statin exposure. Our overall analytic strategy is to use the fits of these models to identify SNPs with strong evidence for association with the bivariate outcome (S,D) (i.e. against the null H_0_), and then among these most associated SNPs to assess the evidence for a statin-response association (H_D_ and H_S+D_) vs. a statin-independent association (H_S_).

This bivariate approach is motivated by the expectation that many genetic variants associated with D will also be associated with S (since to be associated with D but not S a variant would have to have *exactly opposite* effects on Y and X). For these variants, the support for H_S+D_ should generally be greater than the support for H_D_ or H_S_ alone, and so consideration of S and D simultaneously should improve power to detect these kinds of associations. Note that, in particular, this includes SNPs that are associated with post-statin phenotype (Y) but not pre-statin phenotype (X), since these SNPs will be associated with both S and D; similarly for SNPs associated with X but not Y. In principle it would be possible, and probably beneficial, to consider these two particular scenarios explicitly, rather than simply including them in a general search for associations with S and D as we do here. However, the correlation between X and Y complicates this analysis, and so we do not pursue it here.

To assess the relative support for the models H_0_, H_S_, H_D_, and H_S+D_, we used Bayesian methods [18], [24], [25], which have advantages over standard frequentist approaches in this context (reviewed in [24]). For example, from a p-value alone it is difficult to quantify how confident one should be that a given SNP is truly associated with phenotype (e.g. is an association with a p-value of 10^−8^ more likely to be real or a false positive?). In contrast Bayesian methods allow one to assess this confidence directly by providing a posterior probability of association for each SNP. In addition, Bayesian methods can be used to formalize ideas that may be used informally in interpreting the results of frequentist analyses: for example, here we use them to formalize the idea that we may be less skeptical about an observed association with D if it is also accompanied by an association with S.

Our Bayesian analysis involves two steps: first compute a Bayes Factor (BF) for each of the models H_0_, H_S_, H_D_, and H_S+D_, and then combine these BFs with prior probabilities on the models to compute the posterior probability on each model. We now describe each of these steps in more detail.

The BF for each model is given by the ratio of the probability of the observed association data under that model to its probability under H_0_, and provides a natural measure of the strength of the support in the data for that model. For example, a BF of 100 indicates that the association data are 100 times more likely under that model than under the null model. The BF for H_0_ is, by definition, 1. We computed the remaining BFs as follows. Let BF_S_ denote the BF for association with the univariate outcome S, computed using the method from Servin and Stephens [18], and BF_D_ denote the BF for association with the univariate phenotype D. Then the BFs for models H_0_, H_S_, H_D_ and H_S+D_ are 1, BF_S_, BF_D_ and BF_D_ x BF_S_, assuming independence of S and D. Intuitively this approach highlights not only SNPs with strong associations with S (large BF_S_) or with D (large BF_D_), but also SNPs with moderate associations with both S and D (large BF_S_ x BF_D_); this is the main difference between our analysis and a more standard univariate analysis.

To compute the univariate Bayes Factors BF_S_ and BF_D_ we used the prior D2 from Servin and Stephens [18]. This prior allows for both an additive effect (a), and a dominance effect (d) at each SNP, with the expected size of these two effects being controlled by hyperparameters σ_a_ and σ_d_ respectively. To put the majority of weight on near-additive models, while still allowing for dominant/recessive effects, we used σ_d_ = σ_a_/4. To deal with the fact that BFs can be sensitive to choice of σ_a_ we averaged results over multiple values for σ_a_, as suggested in Servin and Stephens [18] and Stephens and Balding [24]. Specifically we used σ_a_ = 0.05, 0.075, 0.1, 0.125, 0.15, 0.2, 0.4 (with equal weight on each). We computed BF_D_ and BF_S_ using the genotype and phenotype data from all individuals combined, and the software BIMBAM and its ability to compute combined BFs from summary data on each study, with posterior mean genotypes at imputed genotypes [19]. This corresponds to assuming that each SNP has the same effect in all studies, and thus to performing a “fixed-effects” combined analysis.

The posterior probabilities on each of the models H_0_, H_S_, H_D_, and H_S+D_ is then computed by combining the BFs with a prior probability distribution on models. Specifically, the posterior probability for each model is computed by multiplying the BF for each model by the model's prior probability, and then normalizing the four resulting products to sum to 1, so that the posterior probabilities on the 4 models sum to 1. In symbols, the posterior probability for model i is given by posterior_i_  =  BF_i_ × prior_i_/Σ_j_ BF_j_ × prior_j_. We used the prior probability distribution: Pr(H_0_) = 1−10^−4^, Pr(H_S_) = 0.9×10^−4^, Pr(H_D_) = 0.01×10^−4^, Pr(H_S+D_) = 0.09×10^−4^. These prior probabilities were chosen to i) place overall prior 10^−4^ on any kind of association for each SNP with a given phenotype, in line with previous suggestions [24], [26], [27]; ii) be substantially more skeptical about associations with D than with S (overall prior on being associated with D = 10^−5^, compared with approximately 10^−4^ on S), and iii) be less skeptical about an association with D if it is also accompanied by an association with S. Posterior probabilities can be sensitive to this choice of prior, particularly posterior probabilities that are not very close to 0 or 1; see Discussion for more on sensitivity. The posterior probabilities on models summarize the overall support for each model, taking account of both the association data and prior beliefs regarding the relative plausibility of the four models. Using these posterior probabilities it is straightforward to assess the overall evidence against the null hypothesis H_0_ (using the sum of the posterior probabilities on H_S_, H_D_ and H_S+D_), and furthermore to partition this overall evidence into evidence for statin-independent associations (posterior probability on H_S_) vs. statin-response associations (sum of the posterior probabilities on H_D_ and H_S+D_).

To allow our results to be more easily compared with standard frequentist analyses, we also used linear regression with S (and respectively D) as the response variable, assuming an additive genetic model at each SNP, to compute p-values for each SNP against the null hypothesis that the SNP is unassociated with S (and respectively D). We report p-values without adjustment for multiple comparisons.

To deal with systematic differences in study population and protocols, we normalized phenotype measurements within each study. Normalized phenotypes for LDLC, TC, Tg and HDLC were derived from raw pre-statin and post-statin measurements following a four-step procedure. First, to limit the influence of outliers, pre-statin and post-statin measurements were rank transformed to a standard normal distribution within each data set (CAP-Stage I, CAP-Stage II, PRINCE-Stage I; PRINCE-Stage II; TNT). Second, derived sum and difference phenotypes (S and D above) were computed from these rank-transformed values. Next, values of S and D were corrected for covariates (log(BMI), age, sex, and smoking status) within each dataset using ordinary least squares regression. Finally, covariate-corrected values of S and D were again rank transformed to normal distributions within each dataset (note that any induced non-zero correlation between the transformed S and D was always negligibly small). We note that within each study the logs of each of the raw lipid measures (both pre- and post- statin) are approximately normally distributed, and so the end result of this extensive normalization is similar to (but not identical to) what would have been obtained by the simpler approach of working with the log-transformed phenotype values, and not performing the rank transformations to normal distributions. We used the rank (rather than log) transformations both to limit the effects of any deviations from normality (e.g. outliers) in the log-transformed phenotypes, and, more importantly, to ensure that there are no distributional differences in tested response variables among studies, so that any differences in allele frequencies among the study samples will not cause spurious associations.

## Results

The characteristics of the three study populations are described in Table 1. Although there are clear differences in clinical characteristics across these populations, our normalization steps should have ensured that systematic differences will not cause false positive associations. However, cryptic population stratification *within* studies could still cause spurious associations [28]. To assess this, we compared the overall distribution of p-values for each tested response (sum, S, and difference, D, for LDLC, TC, Tg and HDLC) with a uniform distribution. QQ plots (Figure S1) showed generally good agreement except in the tail where deviations are expected due to genuine associations. Genomic control inflation factors ranged from 0.99 to 1.03 [29]. In addition, preliminary analyses of the CAP and PRINCE studies that used Principal Components Analysis (PCA) to correct for latent population structure [30] produced similar results to analyses without PCA correction (results not shown). Thus cryptic population stratification does not appear to have a substantive impact on association results in this case.

**Table 1:** Description of Study Populations.

		PARC Populations		
		CAP	PRINCE	TNT
N		592	1360	1976
Gender, N males		313 (52.7%)	1044 (76.9%)	1622 (82.1%)
Age		54.4±12.7	64.7±13.0	62.4±8.3
BMI		27.7±5.5	29.0±5.3	29.0±4.6
Primary CVD (# subjects)		0 (0%)	843 (61.9%)	486 (24.6%)
Smoking (# subjects)		81 (13.6%)	183 (13.4%)	367 (18.6%)
Systolic BP		123.1±16.8	133.4±17.3	132.3±17.3
Diastolic BP		70.7±9.8	79.0±10.1	77.9±9.6
Total Cholesterol				
Untreated		212.2±35.1	215.3±38.6	245.2±29.1
Treated		153.4±27.1	173.5±36.2	174.2±22.5
Change		−58.8±1.0	−41.8±0.8	−71.1±0.5
LDLC				
Untreated		133.0±31.7	131.6±29.1	161.8±22.8
Treated		76.8±22.5	97.7±26.6	97.4±16.0
Change		−56.2±0.9	−33.8±0.6	−64.4±0.4
Triglyceride				
Untreated		127.7±66.9	200.3±131.9	208.7±97.1
Treated		104.4±65.1	165.1±116.6	156.4±71.5
Change		−23.7±1.8	−35.3±2.6	−52.4±1.4
HDLC				
Untreated		53.8±16.3	36.7±10.3	46.8±10.4
Treated		56.0±17.0	38.5±10.6	45.7±10.0
Change		2.3±0.2	1.8±0.2	−1.1±0.1

Abbreviations: CAP, Cholesterol and Pharmacogenetics trial; PRINCE, Pravastatin Inflammation/CRP Evaluation trial; TNT, Treating to New Targets trial.

Table 2 shows regions harboring SNPs that were most strongly associated with sum and/or difference phenotypes from our analysis. This table includes all regions that contained a SNP with >50% posterior probability of being genuinely associated with a phenotype (i.e. <50% posterior probability assigned to H_0_), and associations are presented for the SNP within each region that demonstrated the strongest evidence for association. More comprehensive results are provided in Tables S1, S2, S3, S4, and S5.

**Table 2:** Top variants associated with difference or sum traits for LDLC, total cholesterol, triglyceride, or HDLC.

SNP	Posterior Probability				P-value		MAF*	Chr	Nearest Genes		
	H_0_	H_S_	H_D_	H_(S+D)_	Sum	Diff			Gene Symbols (Distance from variant, kb)		
**Total Cholesterol**											
**rs8014194**	**0.16**	**<0.01**	**0.07**	**0.77**	**0.06**	**1.9×10^−8^**	**0.24** **0.24/0.24/0.25**	**14**	**CLMN (0)**	**FLJ45244 (74)**	**DICER1 (97)**
**LDLC**											
**rs4420638**	**0.30**	**0.36**	**<0.01**	**0.34**	**6.3×10^−7^**	**4.2×10^−3^**	**0.19** **0.19/0.20/0.20**	**19**	**APOCI (0)**	**APOE (10)**	**TOMM40 (16)**
rs646776	0.04	0.92	<0.01	0.04	3.7×10^−8^	0.20	0.190.18/0.20/0.20	1	CELSR2 (0)	PSRC1 (4)	SORT1 (34)
rs7633531	0.13	0.84	<0.01	0.03	1.4×10^−7^	0.37	0.180.19/0.17/0.19	3	C3orf53 (211)	ZCWPW2 (262)	LOC131572 (300)
**Triglyceride**											
**rs1260326**	**<0.01**	**0.83**	**<0.01**	**0.17**	**5.0×10^−15^**	**2.6×10^−2^**	**0.45** **0.46/0.44/0.45**	**2**	**GCKR (0)**	**LOC729823 (11)**	**FNDC4 (13)**
rs964184	<0.01	0.99	<0.01	0.03	1.9×10^−14^	0.46	0.140.14/0.14/0.14	11	ZNF259 (0)	BUD13 (5)	APOA5 (11)
rs9644568	0.06	0.90	<0.01	0.04	4.5×10^−8^	0.47	0.110.11/0.10/0.11	8	SLC18A1 (74)	LPL (104)	ATP6V1B2 (126)
rs1883025	0.35	0.63	<0.01	0.02	6.4×10^−7^	0.35	0.240.23/0.24/0.24	9	ABCA1 (0)	NIPSNAP3B (128)	NIPSNAP3A (142)
**HDLC**											
rs247616	<0.01	0.98	<0.01	0.02	2.5×10^−32^	0.45	0.320.32/0.32/0.33	16	CETP (6)	HERPUD1 (12)	SLC12A3 (42)
rs4775041	0.01	0.96	<0.01	0.03	1.0×10^−8^	0.86	0.250.26/0.24/0.26	15	LIPC (49)	LOC441726 (181)	AQP9 (197)
rs1011685	0.02	0.95	<0.01	0.03	2.1×10^−8^	0.58	0.120.12/0.12/0.13	8	LPL (6)	INTS10 (121)	SLC18A1 (172)

Table includes all regions with strong evidence for association with traits (posterior probability <0.5 for no association). For each region, the SNP with the strongest evidence for association is displayed. H_0_: null model; H_S_: sum model; H_D_: difference model; H_(S+D)_: sum and difference model. SNPs associated with difference traits (H_D_ or H_(S+D)_) are in bold. P-values are uncorrected for multiple testing. Abbreviations: SNP, single nucleotide polymorphism; Diff., different trait; MAF, minor allele frequency; Chr., chromosome.

* MAF listed for total population and then for each individual study (TNT/PRINCE/CAP).

The majority of these putative associations appear to be independent of statin treatment: 8 of the 11 have ≤4% probability of being associated with the difference phenotype D (with or without an accompanying association with the phenotype sum, S). Of these 8 loci, five involve previously-reported associations that have been robustly replicated: HDLC associations with SNPs in or near the genes encoding cholesteryl ester transfer protein (*CETP*), hepatic lipase (*LIPC*), and lipoprotein lipase (*LPL*); triglyceride associations within the region containing apolipoprotein A5, *APOA5*; and an LDLC association with SNPs located in the *CELSR2/PSRC1/SORT1* region of chromosome 1 [11], [16], [31]. Of the other 3 statin-independent associations, the association of triglyceride with rs9644568, located near *LPL*, and rs1883025, located in *ABCA1*, seem the most likely to be genuine, based on the known functions of these genes [32], [33].

**Figure 1:**
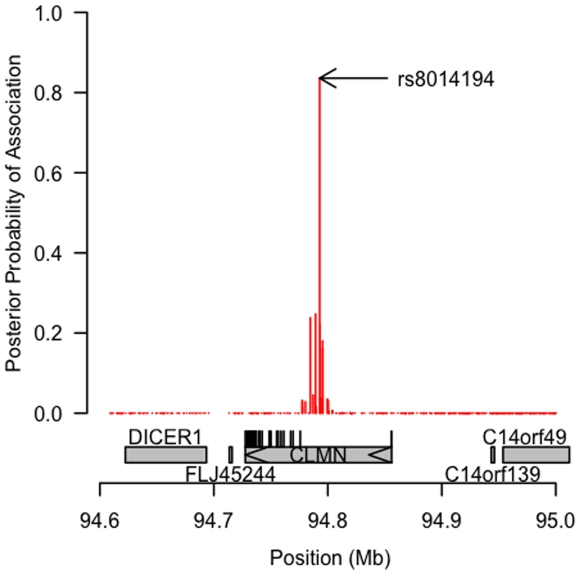
Posterior probability of association with statin-mediated difference trait for total cholesterol at chromosome 14 region. Gene structure indicated below graph. rs8014194, located in *CLMN* intron 1, was the most significantly associated variant.

Our analyses also identified associations with the statin-mediated difference trait. The strongest signal for such an association was between statin-induced change in TC and SNPs within introns 1–2 of the gene encoding calmin (*CLMN*). This region of chromosome 14 has not been previously related to lipid or lipoprotein traits. Multiple SNPs in this region showed association with this trait in both Bayesian and Frequentist analyses (Figure 1). The most strongly-associated SNP was rs8014194. Our Bayesian method assessed this SNP to have an 84% posterior probability of being genuinely associated with statin-mediated change in TC (77% probability of association with both D and S; 7% probability of association with D alone). Consistent with this high posterior probability, the frequentist test for the null hypothesis of no association between this SNP and the difference phenotype D yielded a p-value of 1.9×10^−8^. This SNP was less strongly associated with change in LDLC (posterior probability of association  = 0.16; *P* = 3.9×10^−6^). Examining this SNP's association with the TC difference trait separately within each of the three studies, we observed the strongest association signal in TNT (TNT *P* = 1.2×10^−6^; PRINCE *P* = 4.8×10^−3^; CAP *P* = 0.16, Table S2). On average across studies, carriers with two copies of the minor allele of rs8014194 had 3.0% smaller reductions in total cholesterol than did noncarriers, and variation at rs8014194 explained 1% of the variance in statin-induced changes in total cholesterol.

In addition, the Bayesian analysis yielded two other SNPs showing moderate evidence for being associated with statin-induced phenotype changes. The SNP rs4420638, located in the *APOC1* gene and near *APOE*, was assigned a 34% posterior probability of association with statin-induced change in LDLC. Variation at the *APOE* locus has been associated with statin-mediated lipid response in several studies [4], [13]. In addition rs1260326 located in *GCKR*, was assigned a 17% probability of being associated with statin-induced change in triglyceride. These two putative SNP associations with response phenotypes were highlighted much more strongly in the Bayesian analysis than in the frequentist analysis. The p-values for associations with difference traits of these 2 SNPs were 0.0042 and 0.026 respectively, which puts them well down the list of the top associations. This difference in results between the two methods occurred because both of these SNPs showed an appreciable association with the sum trait, S. In this situation, the Bayesian approach upweights such SNPs because it reinforces the idea that, for a SNP associated with S, a modest association with D is more likely to be genuine than it would be for a SNP that is unassociated with S.

Besides the strongest associations reported here, there were many more variants that showed non-trivial, although far from conclusive, evidence for association with statin response. Two variants were assigned between 10% and 50% probability of being associated with statin-mediated changes in LDLC (Table S1B): rs1431005 located on chromosome 4 (Posterior probability  = 0.13, *P* = 1.8×10^−7^) and rs13390159 located on chromosome 2 (Posterior probability  = 0.17, *P* = 2.1×10^−7^). Both of these variants were less strongly associated with total cholesterol change (Table S1A) and both are located in genomic regions that contain putative genes of unknown function. In addition, two SNPs were assigned 10–50% probability of being associated with statin-mediated change in triglyceride (Table S1C). The SNP rs7584099 was assigned a 22% posterior probability of association with statin-induced change in triglyceride (p = 5.4×10^−7^). This SNP is located 124 Kb from the *ACVR2A* gene (activin A receptor, type IIA), which is a serine/threonine kinase receptor that binds activin and appears to play a role in cellular differentiation and proliferation [34]. The SNP rs174583 was assigned a 10% posterior probability of association with statin-induced change in triglyceride (p = 0.01). This SNP, located in the fatty acid desaturase (*FADS*) gene cluster on chromosome 11 has been identified as strongly associated with baseline triglycerides and HDLC in many studies and was associated with HDLC within this study with a posterior probability of 17% (*P* = 6.8×10^−6^, Table S1D) [12], [31].

Finally, we identified some variants with less strong associations that are near biologically plausible genes. For example, one of the most significant associations with total cholesterol response was a variant located on chromosome 2 near the gene encoding insulin induced gene 2 (*INSIG2*). INSIG2 is involved in sterol-dependent SREBP-mediated regulation of cholesterol metabolism and *INSIG2* has previously been identified within mouse models as a susceptibility gene for total cholesterol [35], [36]. Within this genomic region, the strongest evidence was for association of rs11673900, located 9 kb downstream of *INSIG2* (Table S2A). This SNP was assigned a 5% posterior probability of association with statin-mediated change in total cholesterol (*P* = 5.5×10^−6^).

## Discussion

The genetic contribution to variation in lipid-lowering response to statin treatment appears to be influenced by multiple loci with small individual contributions that are compounded when jointly inherited. Although several loci have been identified in association with statin efficacy using the candidate gene approach, the combined contribution of these genotypes explains a relatively small proportion of the variation in statin LDLC-lowering efficacy [4], [6]. GWA analysis of lipid-lowering response to statin treatment has previously failed to identify novel loci [13] but the statistical power to detect associations was limited by sample size. To address this issue, we have performed a combined analysis on the three statin GWA studies that are currently available. Because each of the three trials tested a different statin, this analysis was specifically designed to identify genetic variation that is associated with statin class effects.

Among the associations identified by our analysis, the most intriguing is the association of rs8014194 with changes in total cholesterol in response to statin treatment. When we examined the association between rs8014194 and change in total cholesterol separately for each trial (TNT, PRINCE, CAP), we observed the strongest association within TNT. While differential ability to detect this association may reflect differences in statistical power across these three studies based on sample size, it may also reflect differences in pharmacological properties (e.g., dose response or statin) or differences in biological mechanism (e.g., lipoprotein homeostasis). This SNP was also less strongly associated with LDL-cholesterol reduction. SNP rs8014194 is located on chromosome 14 within intron 1 of the gene encoding calmin, *CLMN*. The function of calmin is unknown but the protein sequence contains a calponin-like binding domain that is expected to have actin-binding activity [37]. Calmin is highly expressed in several tissues including liver and adipose tissue [38]. Full-length calmin contains a putative transmembrane domain and appears to be localized to the endoplasmic reticulum [37]. Several *CLMN* alternative splice variants encode isoforms that lack the transmembrane domain and appear to be localized to the cytosol [37].

Calmin has not previously been implicated in cholesterol or lipoprotein metabolism nor has *CLMN* variation been associated with any metabolic traits. As a result it is natural to question whether the observed association is likely to be genuine. Our Bayesian analysis assessed the posterior probability of this association being genuine to be 84%. This figure is sensitive to prior assumptions about how likely each individual SNP is to be associated with each statin response trait. Our analysis assumed this to be 1 in 10^5^, corresponding to an a priori assumption that approximately 25 SNPs out of the 2.5 million tested would be genuinely associated with statin response for each phenotype. The data seem broadly consistent with this prior assumption, in that the posterior expected number of such associations ranged from 19 (for HDLC) to 26 (for Tg). However, the data may also be consistent with more conservative assumptions. If one were to select a more conservative prior assumption of 1 in 5×10^5^ (implying approximately 5 genuinely associated SNPs) then the posterior probability for rs8014194 being genuinely associated with total cholesterol response decreases to approximately 50%. Conversely, if one is less skeptical and increases the prior by a factor of 4 (i.e. 100 genuinely-associated SNPs), the posterior increases to over 95%. In a frequentist analysis, degree of skepticism is reflected (implicitly or explicitly) in choice of threshold for “genome-wide significance” [24], [39]. In this case the p-value for association, *P* = 1.9×10^−8^ meets conventional levels for genome-wide significance – for example, it is nominally significant at the 0.05 level after a Bonferroni correction for 2.5 million tests, and exceeds the 5×10^−7^ threshold used by WTCCC in reporting “strongly associated” variants [26]. Thus, there is strong evidence to support the conclusion that rs8014194 is the first novel SNP found through GWAS to be associated with statin response, although definitive confirmation will depend on results from additional statin pharmacogenomic trials. In addition, we identified several SNPs with less strong evidence for association with lipid response to statin treatment including 2 SNPs associated with change in LDLC (posterior probabilities 13% and 17%) located in genomic regions containing putative genes not known to influence lipid metabolism, and 2 SNPs associated with change in Tg including one near *ACVR2A* (posterior probability 22%) and one within the *FADS* locus (posterior probability 10%).

One important issue facing any study of this nature is how to define a “drug-response association”. Naturally, any SNP that is associated with only post-statin levels of the phenotype, and not pre-statin levels, would be considered a drug-response association. However, here we have broadened this definition to include any SNP that has a different effect on average pre- and post- statin phenotype levels. Note that this broader definition depends on the scale on which phenotypes are considered. For example, if we had used the raw scale for pre- and post-statin measures, then a SNP that decreased pre-statin levels by 2 (units) on average and decreased post-statin levels by the same average amount would not count as a statin-response SNP. But if we had used the log-scale for the measures, then the same SNP would become a response association (effectively because 2 units is a different percentage of the average post-statin than of the average pre-statin measures). Our rank-based transformation procedures, which were used to provide strong safeguards against spurious associations due to potential differences in allele frequencies among studies, could potentially complicate interpretation. However, since these rank-based transformations produce phenotypes that are somewhat similar to a log-transformation, we think of our definition of statin-response associations as roughly encompassing SNPs that have a different relative percentage reduction in pre- and post- statin lipid levels. It is also important to note that these definitional subtleties apply only to SNPs that affect both pre- and post- statin levels: SNPs that affect one but not the other would satisfy the definition of statin-response associations whatever scale is used. Nonetheless it remains an open question to what extent SNPs that satisfy our broader definition of statin-response associations have a molecular interaction with the statins themselves, or the genetic pathways they target – but this would be true of any definition, and the fact that most previously-identified loci associated with untreated lipid levels do not show a strong signal for statin-response associations suggests that our definition is a reasonable starting point.

Another issue is whether there might be other, more effective, statistical approaches to identifying statin response associations. It has long been recognized that simply testing groups for association with the change (Y−X) often has low power, because this change generally has a high variance. Our analyses attempt to mediate this problem by instead considering the bivariate outcome (X,Y), or equivalently (Y−X,X+Y), to identify SNPs that are strongly associated with pre- and/or post-statin lipid levels, and then, among these SNPs, attempt to identify the subset of SNPs that appear to have a different effect on Y than X. Intuitively this bivariate approach should help when compared with simply testing (Y−X) alone, because most SNPs that are genuinely associated with (Y−X) are expected to be associated also with Y+X. (e.g. SNPs associated with Y alone would fall into this category). This bivariate approach is particularly helpful to highlight SNPs for which there is complementary evidence for association with both untreated and response traits, as illustrated by the relatively high posterior probabilities assigned to SNPs near *APOE* and *GCKR* in Table 2 for association with statin-related change of LDLC and Tg, respectively. Another source of information that one might incorporate into these analyses to help improve power is the increasing amount of data from other genetic association studies identifying SNPs associated with untreated lipid levels [9], [10], [12], [15], [16], [31]. Within our Bayesian analysis this could be easily achieved by placing a much lower prior probability on the null hypothesis H_0_ for these SNPs with prior evidence for association. However, one could also incorporate this information into a frequentist analysis: to give just a simple example, in a standard frequentist genome scan testing SNPs for association with (Y−X), one could use a less stringent significance threshold for those SNPs already known from other studies to affect untreated lipid phenotype levels. Of course, choice of appropriate threshold is a tricky problem, and one possible reason to prefer the Bayesian approach.

The statistical approach we have taken here differs from that taken in previous analysis of the TNT data [13], which tested for association between genetic variants (G) and post-statin phenotype measures (Y), controlling for pre-statin phenotype measures (X). While this type of analysis, sometimes referred to as ANCOVA, is standard for identifying group treatment effects in a randomized controlled trial, it must be applied with caution in the setting of genetic association studies. This is because this ANCOVA test is aimed at rejecting the null hypothesis that Y is conditionally independent of G given X, and rejecting this hypothesis in a genetic association study does not necessarily imply that G is associated with statin response. For example, if a genetic variant G affects average pre- and post- statin lipid levels in the same way, then by our definition it would not be a statin-response association, but the ANCOVA test would tend to give a significant result. This claim is easily verified by simulation (Figure S2 and File S1). Note that the situation here is fundamentally different from a randomized controlled trial, because the randomization is usually designed to ensure that baseline measures (X) are unassociated with the groups G being tested, in which case G will be associated with the response (Y−X) if and only if it is associated with Y controlling for X, and the ANCOVA test is preferred due to its greater power [40]. This said, since one plausible type of statin-response association is a SNP that is associated with Y and not X, it would seem fruitful to take this into account in the analysis, and with additional work this could be incorporated into our Bayesian framework as an explicit additional hypothesis.

Finally regarding statistical methodology, we chose here to perform a “fixed effects” combined analysis that assumes the effect sizes are the same across studies, despite the fact that the studies are clearly highly heterogeneous. We did this because we judged that, due to limited sample size within each study, power would be limited to detect any effects that did not appear consistent across studies. Further, separate analyses of each study failed to yield convincing evidence for loci with strong, but possibly different, signals in more than one study. We note that although heterogeneity across studies clearly results in model mis-specification (and hence potential loss of power) under the alternative, the null model remains unaffected by this heterogeneity (after our normalization procedures ensuring that the phenotypes have the same distribution in each study) and so, under the null, heterogeneity should not cause false positive associations to be detected by our analysis.

There were several limitations of this study. Because different statins were tested in each of the three trials used for this combined analysis, the results cannot be extrapolated to individual statins. This may explain why no associations were observed with genes involved in pharmacokinetic handling of statins. Separate analyses of each study yielded no SNPs with high probability of being associated with statin response phenotypes, presumably due to limits in statistical power. There were also several major differences in study populations across these trials, the major one being the inclusion in PRINCE and TNT, but not CAP, of individuals with documented CHD or CVD events. In addition, untreated LDLC concentrations were higher in TNT than in CAP or PRINCE, and this variation across study populations in underlying CVD risk may influence genetic contribution to statin response. Moreover, as noted above, to exclude individuals with poor drug compliance, inclusion in TNT was limited to those who achieved LDLC≤130 mg/dL with atorvastatin treatment (4 weeks, 10 mg/day), and this likely led to underrepresentation of genotypes associated with attenuated statin response. Although we used imputation methods to maximize genomic coverage for these analyses, this method is ultimately limited by the genomic coverage of the underlying genotype panels. In particular, variation at genomic regions with poor coverage, such as the *APOE* locus, cannot be completely described through imputation. Thus, the relatively modest posterior probability assigned to SNPs near the *APOE* locus in this case may reflect the relatively small number of individuals for whom genotypes were measured. In addition, the SNPs at the HMG-CoA reductase (*HMGCR*) locus that were identified in the PRINCE and CAP populations to be associated with statin- mediated LDLC response [7], [22] were not genotyped in HapMap and, therefore, were not represented in this analysis. Furthermore, rarer SNPs or haplotypes that may have large effect sizes could not be assessed. Finally a major limitation is the exclusion of individuals not of European ancestry. Although we have incorporated all available published pharmacogenomic studies of statin efficacy into our combined analysis, this study is probably still underpowered and identification of variants with statistically meaningful association to statin efficacy will require analyses in expanded populations once GWA data from additional trials becomes available. Despite this, results from this and other studies suggest that no single SNP will describe more than ∼3% of the variance observed in lipid-lowering response to statin treatment.

In summary, using Bayesian imputation-based analysis on a combined population derived from the three currently available statin GWA trials, we have identified a new candidate gene, calmin, that may modulate statin-mediated changes in total cholesterol and LDLC. This is the first report of a variant associated with statin efficacy that was identified by GWAS and its validation awaits functional analyses and replication in additional statin trials.

## Supporting Information

Figure S1 Q-Q plots for sum traits (A, total cholesterol; B, LDL-cholesterol; C, HDL-cholesterol; D, triglyceride) and for difference traits (E, total cholesterol; F, LDL-cholesterol; G, HDL-cholesterol; H, triglyceride).
(9.78 MB TIF)

Figure S2 Simulated illustration of behavior of the ANCOVA test when applied to SNPs that have the same effect on both pre- and post- exposure measurements. (A) histogram of p values from the ANCOVA test. (B) histogram of p values from tests of the difference Y-X against genotype (shown for comparison only, not to advocate this test). The R code used to produce this Figure is given in File S1 (Supplementary Methods). The non-uniform p values in the top plot indicate that the ANCOVA test can tend to give significant results for SNPs that affect both pre- and post- exposure measures in the same way. As a result, a significant ANCOVA test does not necessarily indicate a statin-dependent association.
(9.41 MB TIF)

File S1 Supplementary Methods: R code to do simulations to illustrate that ANCOVA test can produce non-uniform p values when a SNP has the same effect on both pre- and post- exposure measurements as shown in Figure S2.
(0.02 MB DOC)

Table S1 Extended GWAS associations. For each trait (A, total cholesterol; B, LDL-Cholsterol; C, HDL-Cholesterol; D, triglyceride) the regions with moderate evidence for association (posterior probability of 0.5 to 0.89 for the null hypothesis, H0) are represented by the SNP within that region that showed the strongest evidence for association. Associations with difference traits are displayed in bold. H0: null model; HS: sum model; HD: difference model; H(S+D): sum and difference model. Abbreviations: SNP, single nucleotide polymorphism; Diff., different trait; MAF, minor allele frequency; Chr., chromosome.
(0.11 MB DOC)

Table S2 Single nucleotide polymorphisms (SNPs) associated with difference or sum traits for total cholesterol with a posterior probability greater than 1%. SNPs are listed by reference SNP accession ID (rs#) and are ordered by chromosome number and location on each chromosome. Posterior probabilities calculated by Bayesian analysis are listed for H0, HS, HD, HS+D followed by p-values and effect sizes as calculated by frequentist statistics for the sum and difference traits. Major and minor alleles are listed. Abbreviations: MAF, minor allele frequency; chr, chromosome; GENE1-3, genes closest to variant; dist1-3, distance of gene1-3 to variant; log10BF, log(base 10) transformed bayes factor for: S, sum trait in total population; S-TNT, sum trait in TNT population; S-PRINCE, sum trait in PRINCE population; S-CAP, sum trait in CAP population; D, difference trait in total population; D-TNT, difference trait in TNT population; D-PRINCE, difference trait in PRINCE population; D-CAP, difference trait in CAP population; P-values from individual populations are also listed for each SNP. A complete results list for all ∼2.5 million sites is available online at http://stephenslab.uchicago.edu/publications.html.
(0.68 MB XLS)

Table S3 Single nucleotide polymorphisms (SNPs) associated with difference or sum traits for LDL-cholesterol with a posterior probability greater than 1%. SNPs are listed by reference SNP accession ID (rs#) and are ordered by chromosome number and location on each chromosome. Posterior probabilities calculated by Bayesian analysis are listed for H0, HS, HD, HS+D followed by p-values and effect sizes as calculated by frequentist statistics for the sum and difference traits. Major and minor alleles are listed. Abbreviations: MAF, minor allele frequency; chr, chromosome; GENE1-3, genes closest to variant; dist1-3, distance of gene1-3 to variant; log10BF, log(base 10) transformed bayes factor for: S, sum trait in total population; S-TNT, sum trait in TNT population; S-PRINCE, sum trait in PRINCE population; S-CAP, sum trait in CAP population; D, difference trait in total population; D-TNT, difference trait in TNT population; D-PRINCE, difference trait in PRINCE population; D-CAP, difference trait in CAP population; P-values from individual populations are also listed for each SNP. A complete results list for all ∼2.5 million sites is available online at http://stephenslab.uchicago.edu/publications.html.
(0.67 MB XLS)

Table S4 Single nucleotide polymorphisms (SNPs) associated with difference or sum traits for HDL-cholesterol with a posterior probability greater than 1%. SNPs are listed by reference SNP accession ID (rs#) and are ordered by chromosome number and location on each chromosome. Posterior probabilities calculated by Bayesian analysis are listed for H0, HS, HD, HS+D followed by p-values and effect sizes as calculated by frequentist statistics for the sum and difference traits. Major and minor alleles are listed. Abbreviations: MAF, minor allele frequency; chr, chromosome; GENE1-3, genes closest to variant; dist1-3, distance of gene1-3 to variant; log10BF, log(base 10) transformed bayes factor for: S, sum trait in total population; S-TNT, sum trait in TNT population; S-PRINCE, sum trait in PRINCE population; S-CAP, sum trait in CAP population; D, difference trait in total population; D-TNT, difference trait in TNT population; D-PRINCE, difference trait in PRINCE population; D-CAP, difference trait in CAP population; P-values from individual populations are also listed for each SNP. A complete results list for all ∼2.5 million sites is available online at http://stephenslab.uchicago.edu/publications.html.
(0.71 MB XLS)

Table S5 Single nucleotide polymorphisms (SNPs) associated with difference or sum traits for triglyceride with a posterior probability greater than 1%. SNPs are listed by reference SNP accession ID (rs#) and are ordered by chromosome number and location on each chromosome. Posterior probabilities calculated by Bayesian analysis are listed for H0, HS, HD, HS+D followed by p-values and effect sizes as calculated by frequentist statistics for the sum and difference traits. Major and minor alleles are listed. Abbreviations: MAF, minor allele frequency; chr, chromosome; GENE1-3, genes closest to variant; dist1-3, distance of gene1-3 to variant; log10BF, log(base 10) transformed bayes factor for: S, sum trait in total population; S-TNT, sum trait in TNT population; S-PRINCE, sum trait in PRINCE population; S-CAP, sum trait in CAP population; D, difference trait in total population; D-TNT, difference trait in TNT population; D-PRINCE, difference trait in PRINCE population; D-CAP, difference trait in CAP population; P-values from individual populations are also listed for each SNP. A complete results list for all ∼2.5 million sites is available online at http://stephenslab.uchicago.edu/publications.html.
(0.92 MB XLS)
