# Supplementary figures and images for: Genome-Wide Association of Lipid-Lowering Response to Statins in Combined Study Populations
Source: PLoS One. 2010 Mar 22;5(3):e9763. doi: 10.1371/journal.pone.0009763 (PMC2842298; doi:10.1371/journal.pone.0009763)

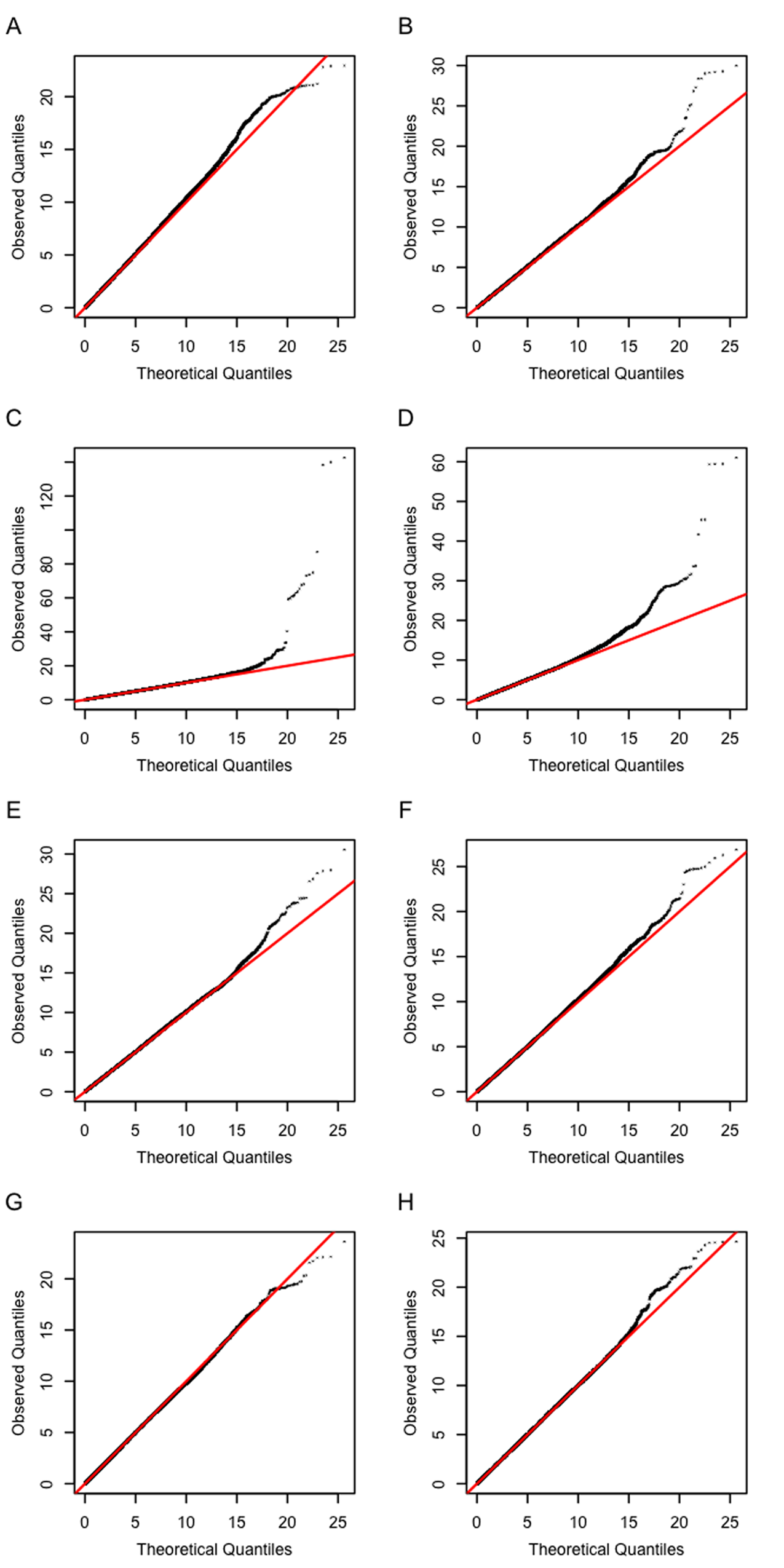

Supplement: Figure S1 — Q-Q plots for sum traits (A, total cholesterol; B, LDL-cholesterol; C, HDL-cholesterol; D, triglyceride) and for difference traits (E, total cholesterol; F, LDL-cholesterol; G, HDL-cholesterol; H, triglyceride). (9.78 MB TIF) [file pone.0009763.s001.tif]

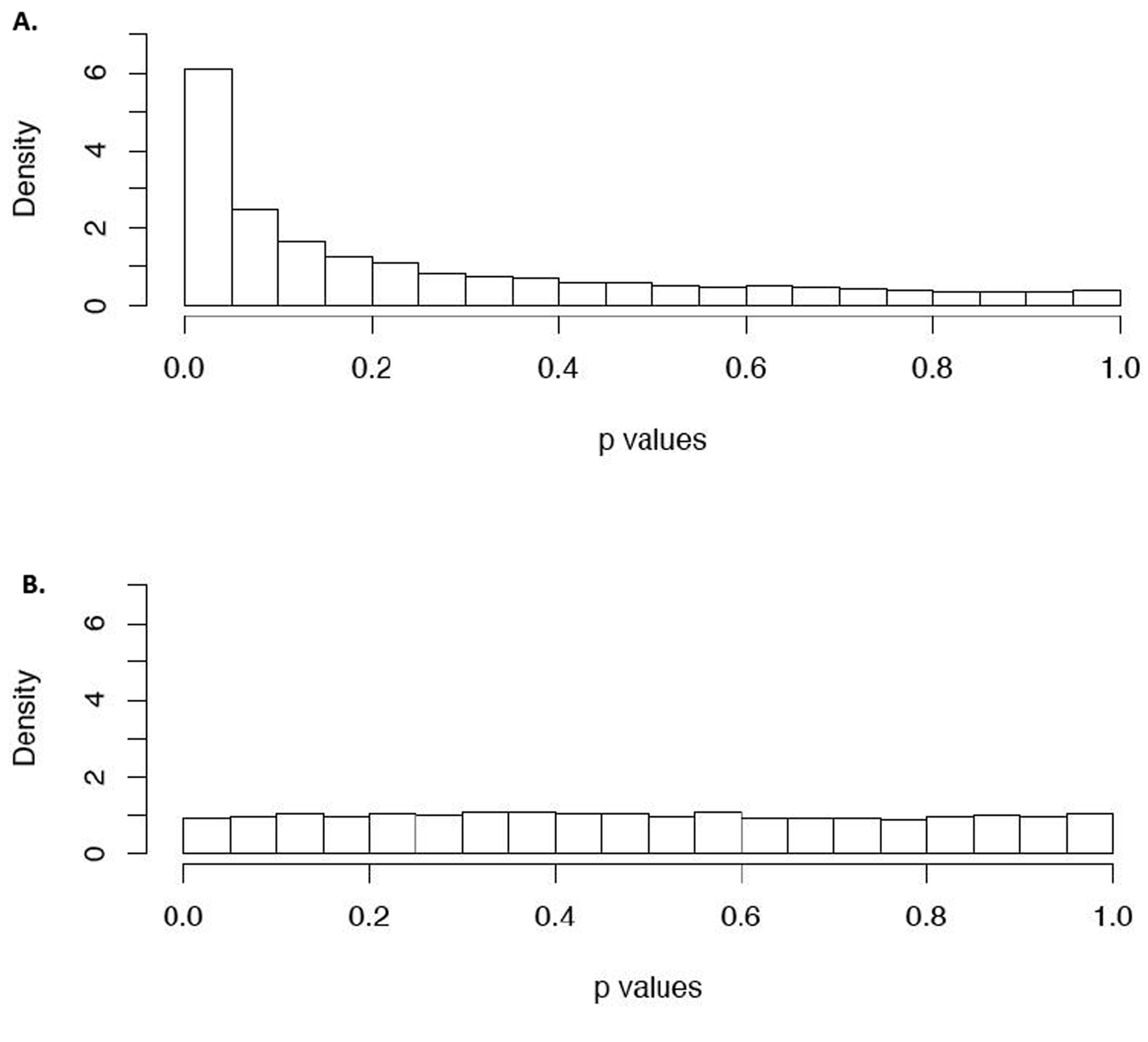

Supplement: Figure S2 — Simulated illustration of behavior of the ANCOVA test when applied to SNPs that have the same effect on both pre- and post- exposure measurements. (A) histogram of p values from the ANCOVA test. (B) histogram of p values from tests of the difference Y-X against genotype (shown for comparison only, not to advocate this test). The R code used to produce this Figure is given in File S1 (Supplementary Methods). The non-uniform p values in the top plot indicate that the ANCOVA test can tend to give significant results for SNPs that affect both pre- and post- exposure measures in the same way. As a result, a significant ANCOVA test does not necessarily indicate a statin-dependent association. (9.41 MB TIF) [file pone.0009763.s002.tif]
