# Supplementary material for: Genome-Wide Association of Lipid-Lowering Response to Statins in Combined Study Populations
Source: PLoS One. 2010 Mar 22;5(3):e9763. doi: 10.1371/journal.pone.0009763 (PMC2842298; doi:10.1371/journal.pone.0009763)
Supplement: File S1 — Supplementary Methods: R code to do simulations to illustrate that ANCOVA test can produce non-uniform p values when a SNP has the same effect on both pre- and post- exposure measurements as shown in Figure S2. (0.02 MB DOC) [file pone.0009763.s003.doc]

**Supplementary Methods: R code to do simulations to illustrate that ANCOVA test can produce non-uniform p values when a SNP has the same effect on both pre- and post- exposure measurements.** (The uniform p values from tests of difference are also shown for contrast).

Nsim = 10000 # number of simulations

Nind = 1000 # number of individuals

p1 = rep(0,Nsim) # set up vector to store p values from ancova test

p2 = rep(0,Nsim) # set up vector to store p values from difference test

for(i in 1:Nsim){

g = rbinom(Nind,2,0.2) # simulate genotypes as binomial, with frequency 0.2

mu = rnorm(Nind,0,1) # simulate individual-specific means, common to

pre and post-exposure measures

x = mu+ 0.2 * g + rnorm(Nind,0,1) #set up x and y so that genotype g

has same effect (0.2) on each

y = mu+ 0.2 * g + rnorm(Nind,0,1)

p1[i] = summary(lm(y~g+x))$coeff[2,4] # p value for coefficient of g

from regression of y against g and x (ANCOVA)

p2[i] = summary(lm(y-x~g))$coeff[2,4] # p value for coefficient of g

from regression of y-x against g

}

pdf("hist.pvalues.pdf")

par(mfcol=c(2,1)) #R command to put 2 plots on 1 page

hist(p1,main="p values from ANCOVA test",ylim=c(0,7),prob=T,xlab="p values")

hist(p2,main="p values from difference test",ylim=c(0,7),prob=T,xlab="p values")

dev.off()
