# Supplementary material for: Genome-Wide Association of Lipid-Lowering Response to Statins in Combined Study Populations
Source: PLoS One. 2010 Mar 22;5(3):e9763. doi: 10.1371/journal.pone.0009763 (PMC2842298; doi:10.1371/journal.pone.0009763)
Supplement: Table S1 — Extended GWAS associations. For each trait (A, total cholesterol; B, LDL-Cholsterol; C, HDL-Cholesterol; D, triglyceride) the regions with moderate evidence for association (posterior probability of 0.5 to 0.89 for the null hypothesis, H0) are represented by the SNP within that region that showed the strongest evidence for association. Associations with difference traits are displayed in bold. H0: null model; HS: sum model; HD: difference model; H(S+D): sum and difference model. Abbreviations: SNP, single nucleotide polymorphism; Diff., different trait; MAF, minor allele frequency; Chr., chromosome. (0.11 MB DOC) [file pone.0009763.s004.doc]

**Table S1A. Total Cholesterol**

| **SNP** | **Posterior Probability** | | | | **P-value** | | **MAF** | **Chr.** | **Nearest Genes** | | |
| --- | --- | --- | --- | --- | --- | --- | --- | --- | --- | --- | --- |
|  | **H_0_** | **H_S_** | **H_D_** | **H_(S+D)_** | **Sum** | **Diff** |  |  | **Gene Symbols (Distance from variant, kb)** | | |
| rs6658356 | 0.68 | 0.30 | <0.01 | 0.01 | 2.1e-06 | 0.58 | 0.06 | 1 | PRDM16 (0) | ARHGEF16 (100) | MEGF6 (126) |
| rs2053302 | 0.69 | 0.30 | <0.01 | 0.01 | 3.7e-06 | 0.36 | 0.18 | 1 | FAM5C (395) | LOC647132 (872) |  |
| rs646776 | 0.75 | 0.24 | <0.01 | <0.01 | 4.2e-06 | 0.52 | 0.19 | 1 | CELSR2 (0) | PSRC1 (4) | SORT1 (34) |
| rs17540621 | 0.75 | 0.24 | <0.01 | 0.01 | 1.8e-06 | 0.69 | 0.06 | 2 | TTC7A (0) | MCFD2 (87) | FLJ40172 (126) |
| rs6070116 | 0.77 | 0.21 | <0.01 | 0.02 | 4.4e-06 | 0.78 | 0.25 | 20 | HMG1L1 (19) | CTCFL (28) | RBM38 (60) |
| rs10950821 | 0.85 | 0.15 | <0.01 | <0.01 | 7.4e-06 | 0.86 | 0.26 | 7 | LOC402641 (17) | ABCB5 (40) | SP8 (175) |
| rs6588480 | 0.85 | 0.14 | <0.01 | <0.01 | 7.6e-06 | 0.32 | 0.15 | 1 | GLIS1 (0) | DMRTB1 (45) | FLJ40434 (73) |
| rs13150348 | 0.86 | 0.14 | <0.01 | <0.01 | 1.2e-05 | 0.63 | 0.25 | 4 | LOC644042 (7) | F11 (48) | KLKB1 (78) |
| rs729397 | 0.86 | 0.14 | <0.01 | <0.01 | 7.9e-06 | 0.49 | 0.26 | 10 | LOC727878 (9) | LOC728209 (268) | LOC441546 (577) |
| rs10893850 | 0.86 | 0.13 | <0.01 | 0.02 | 1.3e-05 | 0.045 | 0.27 | 11 | ETS1 (127) | FLI1 (362) | LOC387820 (375) |
| rs35964523 | 0.87 | 0.12 | <0.01 | 0.01 | 7.9e-06 | 0.79 | 0.42 | 3 | CNTN6 (236) | LOC402123 (239) | CHL1 (448) |
| rs1883025 | 0.87 | 0.12 | <0.01 | 0.01 | 9.8e-06 | 0.43 | 0.24 | 9 | ABCA1 (0) | NIPSNAP3B (128) | NIPSNAP3A (142) |
| rs6554906 | 0.88 | 0.11 | <0.01 | <0.01 | 1.5e-05 | 0.52 | 0.27 | 5 | FBXL7 (0) | LOC441061 (129) | LOC391741 (486) |
| rs10270805 | 0.88 | 0.11 | <0.01 | <0.01 | 9.3e-06 | 0.20 | 0.07 | 7 | SOSTDC1 (1) | LOC442511 (39) | LOC729920 (84) |
| rs4680784 | 0.89 | 0.10 | <0.01 | <0.01 | 1.2e-05 | 0.67 | 0.17 | 3 | C3orf53 (214) | ZCWPW2 (265) | LOC131572 (297) |
| rs6465088 | 0.89 | 0.10 | <0.01 | <0.01 | 1.3e-05 | 0.07 | 0.27 | 7 | GRM3 (0) | KIAA1324L (39) | DMTF1 (311) |
| rs9929488 | 0.89 | 0.10 | <0.01 | <0.01 | 1.6e-05 | 0.09 | 0.29 | 16 | CETP (0) | HERPUD1 (21) | SLC12A3 (51) |
| rs2824443 | 0.89 | 0.10 | <0.01 | <0.01 | 2.3e-05 | 0.98 | 0.19 | 21 | C21orf114 (22) | BTG3 (70) | C21orf91 (110) |

Table S1B. LDL-Cholesterol

| **SNP** | **Posterior Probability** | | | | **P-value** | | **MAF** | **Chr.** | **Nearest Genes** | | |
| --- | --- | --- | --- | --- | --- | --- | --- | --- | --- | --- | --- |
|  | **H_0_** | **H_S_** | **H_D_** | **H_(S+D)_** | **Sum** | **Diff** |  |  | **Gene Symbols (Distance from variant, kb)** | | |
| **rs13390159** | **0.83** | **<0.01** | **0.04** | **0.13** | **0.35** | **2.1e-07** | **0.16** | **2** | **ASB18 (6)** | **LOC728087 (70)** | **IQCA (76)** |
| **rs8014194** | **0.84** | **<0.01** | **<0.01** | **0.16** | **0.0092** | **3.9e-06** | **0.24** | **14** | **CLMN (0)** | **FLJ45244 (74)** | **DICER1 (97)** |
| **rs1431005** | **0.87** | **<0.01** | **0.05** | **0.09** | **0.59** | **1.8e-07** | **0.4** | **4** | **LOC644282 (17)** | **LOC644325 (322)** | **MRPS36P2 (523)** |
| rs10746514 | 0.51 | 0.47 | <0.01 | <0.01 | 1.3e-06 | 0.23 | 0.42 | 1 | DISC1 (69) | SIA1L2 (288) | DISC2 (292) |
| rs4684585 | 0.52 | 0.45 | <0.01 | <0.01 | 8.9e-07 | 0.60 | 0.43 | 3 | LOC402123 (224) | CNTN6 (251) | CHL1 (433) |
| rs12527253 | 0.60 | 0.38 | <0.01 | <0.01 | 1.2e-06 | 0.83 | 0.34 | 6 | RNGTT (26) | ACTBP8 (307) | LOC644119 (318) |
| rs13148903 | 0.70 | 0.29 | <0.01 | <0.01 | 2.7e-06 | 0.58 | 0.25 | 4 | LOC644042 (7) | F11 (48) | KLKB1 (78) |
| rs12643250 | 0.77 | 0.22 | <0.01 | <0.01 | 1.9e-05 | 0.97 | 0.35 | 4 | RHOH (17) | CHRNA9 (93) | N4BP2 (106) |
| rs7900909 | 0.84 | 0.15 | <0.01 | <0.01 | 7.9e-06 | 0.39 | 0.15 | 10 | ANXA2P3 (137) | LOC645084 (519) | RPL7AP1 (785) |
| rs541041 | 0.85 | 0.14 | <0.01 | <0.01 | 7.7e-06 | 0.91 | 0.16 | 2 | APOB (28) | FLJ21820 (262) | GDF7 (414) |
| rs10266483 | 0.86 | 0.14 | <0.01 | <0.01 | 7.9e-06 | 0.17 | 0.35 | 7 | ZNF679 (6) | LOC728927 (59) | LOC442320 (90) |
| rs33153 | 0.89 | 0.11 | <0.01 | <0.01 | 1.4e-05 | 0.29 | 0.3 | 12 | LOC390299 (31) | LOC645485 (50) | C1QDC1 (76) |

**Table S1C**. HDL-cholesterol

| **SNP** | **Posterior Probability** | | | | **P-value** | | **MAF** | **Chr.** | **Nearest Genes** | | |
| --- | --- | --- | --- | --- | --- | --- | --- | --- | --- | --- | --- |
|  | **H_0_** | **H_S_** | **H_D_** | **H_(S+D)_** | **Sum** | **Diff** |  |  | **Gene Symbols (Distance from variant, kb)** | | |
| rs10091038 | 0.67 | 0.32 | <0.01 | 0.01 | 2.8e-06 | 0.18 | 0.43 | 8 | LOC646909 (129) | DUSP4 (152) | KIF13B (240) |
| rs10123041 | 0.81 | 0.18 | <0.01 | <0.01 | 5.9e-06 | 0.96 | 0.16 | 9 | SLC28A3 (0) | RMI1 (303) | LOC729388 (325) |
| rs1535 | 0.83 | 0.17 | <0.01 | <0.01 | 6.8e-06 | 0.56 | 0.32 | 11 | FADS2 (0) | FADS1 (14) | FEN (33) |
| rs11641231 | 0.85 | 0.14 | <0.01 | 0.01 | 8.3e-06 | 0.07 | 0.23 | 16 | LOC401864 (87) | LOC283904 (140) | LOC729464 (148) |
| rs16839962 | 0.86 | 0.13 | <0.01 | <0.01 | 8.7e-06 | 0.48 | 0.13 | 2 | NR4A2 (256) | GPD2 (355) | LOC728038 (367) |
| rs1431315 | 0.88 | 0.11 | <0.01 | <0.01 | 1.2e-05 | 0.41 | 0.11 | 17 | ANKFN1 (80) | PCTP (297) | TMEM100 (351) |

**Table S1D. Tri**glyceride

| **SNP** | **Posterior Probability** | | | | **P-value** | | **MAF** | **Chr** | **Nearest Genes** | | |
| --- | --- | --- | --- | --- | --- | --- | --- | --- | --- | --- | --- |
|  | **H_0_** | **H_S_** | **H_D_** | **H_(S+D)_** | **Sum** | **Diff** |  |  | **Gene Symbols (Distance from variant, kb)** | | |
| **rs174583** | **0.63** | **0.27** | **<0.01** | **0.10** | **2.6e-06** | **0.011** | **0.34** | **11** | **FADS2 (0)** | **FADS1 (25)** | **FADS3 (31)** |
| **rs7584099** | **0.78** | **<0.01** | **0.03** | **0.19** | **0.075** | **5.4e-07** | **0.41** | **2** | **ACVR2A (124)** | **ORC4L (213)** | **LOC647065 (299)** |
| rs16909449 | 0.56 | 0.42 | <0.01 | 0.02 | 1.2e-06 | 0.45 | 0.25 | 9 | MIRN147 (146) | CDK5RAP2 (290) | MEGF9 (502) |
| rs7979575 | 0.61 | 0.35 | <0.01 | 0.04 | 1.8e-06 | 0.089 | 0.16 | 12 | SOX5 (0) | FLJ32894 (441) | BCAT1 (685) |
| rs16883019 | 0.64 | 0.33 | <0.01 | 0.02 | 6.6e-07 | 0.54 | 0.04 | 6 | LOC729105 (40) | ID4 (129) | MBOAT1 (131) |
| rs11932593 | 0.68 | 0.31 | <0.01 | 0.01 | 2.4e-06 | 0.25 | 0.48 | 7 | LOC729920 (0) | LOC442511 (57) | SOSTDC1 (120) |
| rs2901331 | 0.78 | 0.21 | <0.01 | <0.01 | 5.5e-06 | 0.27 | 0.31 | 2 | CNTNAP5 (0) | NA (NA) | NA (NA) |
| rs2954038 | 0.82 | 0.18 | <0.01 | <0.01 | 7.1e-06 | 0.21 | 0.32 | 8 | TRIB1 (57) | NSMCE2 (128) | KIAA0196 (411) |
| rs1993302 | 0.84 | 0.15 | <0.01 | <0.01 | 5.3e-05 | 0.99 | 0.39 | 1 | FAM5C (664) | NA (NA) | NA (NA) |
| rs2305929 | 0.84 | 0.15 | <0.01 | <0.01 | 1.3e-05 | 0.72 | 0.2 | 2 | BRE (0) | RBKS (1) | MRPL33 (111) |
| rs1218282 | 0.85 | 0.14 | <0.01 | 0.02 | 5.3e-06 | 0.11 | 0.06 | 13 | LOC390415 (0) | LOC647298 (30) | C13orf7 (223) |
| rs9305406 | 0.85 | 0.14 | <0.01 | <0.01 | 8.1e-06 | 0.58 | 0.2 | 21 | GRIK1 (74) | CLDN17 (152) | CLDN8 (200) |
| rs10221833 | 0.85 | 0.14 | <0.01 | <0.01 | 6.3e-06 | 0.44 | 0.09 | 2 | COBLL1 (0) | LOC728184 (32) | GRB14 (99) |
| rs3757057 | 0.85 | 0.14 | <0.01 | <0.01 | 3.8e-06 | 0.69 | 0.05 | 6 | BAI3 (0) | LMBRD1 (312) | COL19A1 (502) |
| rs12641400 | 0.89 | 0.11 | <0.01 | <0.01 | 1.4e-05 | 0.19 | 0.11 | 4 | LOC152742 (13) | LOC391636 (121) | LOC644868 (466) |
| rs2023844 | 0.89 | 0.10 | <0.01 | <0.01 | 1.1e-05 | 0.32 | 0.07 | 7 | HOXA13 (4) | HOXA11S (14) | HOXA11 (18) |
| rs4366940 | 0.89 | 0.10 | <0.01 | <0.01 | 1.2e-05 | 0.15 | 0.07 | 2 | ANTXR1 (0) | GKN1 (59) | GDDR (87) |
| rs4513114 | 0.89 | 0.11 | <0.01 | <0.01 | 1.9e-05 | 0.97 | 0.43 | 16 | CNTNAP4 (307) | MON1B (324) | ADAMTS18 (416) |
| rs227761 | 0.89 | 0.11 | <0.01 | <0.01 | 1.1e-05 | 0.89 | 0.5 | 16 | IQCK (0) | GPRC5B (9) | LOC400506 (132) |
